# Monomorphic Epitheliotropic Intestinal T-cell Lymphoma With Initial Pulmonary Symptoms: A Case Report

**DOI:** 10.7759/cureus.79209

**Published:** 2025-02-18

**Authors:** Amy Yeung, TaeHoon Kim, Jason Suh, Metin Taskin, Sita Chokhavatia

**Affiliations:** 1 Department of Internal Medicine, Valley Health System/Icahn School of Medicine at Mount Sinai, Paramus, USA; 2 Department of Hematology and Oncology, Valley Health System/Icahn School of Medicine at Mount Sinai, Paramus, USA; 3 Department of Pathology, Valley Health System/Icahn School of Medicine at Mount Sinai, Paramus, USA; 4 Department of Gastroenterology, Valley Health System/Icahn School of Medicine at Mount Sinai, Paramus, USA

**Keywords:** colonic inflammation, gastrointestinal lymphomas, malignancy, pulmonary, t-cell lymphoma

## Abstract

Monomorphic epitheliotropic intestinal T-cell lymphoma (MEITL) is a rare and aggressive T-cell lymphoma that primarily affects the small intestine. Our case highlights a 60-year-old woman who initially presented with dyspnea, cough, and hemoptysis, followed by gastrointestinal symptoms during hospitalization. Imaging revealed cavitary lung masses, and biopsies confirmed MEITL with a TCR-delta+, CD8+, CD56+, and CD103+ immunophenotype. Endoscopy revealed duodenal nodularity and gastric ulcers; colonoscopy showed diffuse colonic inflammation. This case emphasizes the rare pulmonary involvement in MEITL and expands the limited literature on its extraintestinal manifestations. Clinicians should maintain a high index of suspicion for MEITL in patients presenting with both pulmonary and concurrent gastrointestinal complaints. Early recognition and biopsy-driven diagnosis are critical for timely intervention in this aggressive malignancy.

## Introduction

Monomorphic epitheliotropic intestinal T-cell lymphoma (MEITL) is a rare and highly aggressive subtype of T-cell lymphoma. In the 2016 World Health Organization (WHO) classification, it was reclassified from type II enteropathy-associated T-cell lymphoma due to its lack of association with celiac disease [1]. This disease typically occurs in the sixth decade, with a nearly equal sex distribution [2]. It consists of monomorphic small-to-medium-sized cells that express CD8 and CD56 and primarily involves the small intestine and more uncommonly, the stomach and colon [2,3]. The most common clinical manifestations include abdominal pain, malnutrition, and even intestinal obstruction [3]. However, diagnosis is challenging due to its non-specific symptoms and the low specificity of many diagnostic approaches. Definitive diagnosis relies on biopsy and histopathological evaluation. Although MEITL primarily originates in the intestine, pulmonary involvement is rare but has been documented in a few case reports [2]. We present an unusual presentation of MEITL in a 60-year-old woman who initially presented with pulmonary symptoms followed by the onset of gastrointestinal symptoms during hospitalization.

## Case presentation

A 60-year-old previously healthy Filipino woman presented to the hospital with a six-month history of progressively worsening dyspnea and cough that began after a root canal. Her symptoms initially improved with azithromycin. An outpatient evaluation included a chest computed tomography (CT) scan which revealed a 10.0 x 6.3 cm cavitary lesion in the left upper lobe and a 2.2 x 2.0 cm lesion in the right lung. Two months ago, she was previously admitted to a community hospital where she was diagnosed with necrotizing pneumonia and treated with piperacillin-tazobactam and cefuroxime. She now presents with progressive cough, hemoptysis, and a 20-pound unintentional weight loss. Initial examination on admission was unremarkable. On the fifth day of her admission, she developed persistent non-bloody loose stools and nausea. Notably, her medical history included a normal colonoscopy conducted three months prior to the onset of these symptoms.

On the fifth day of admission, physical examination revealed an afebrile, ill-appearing woman with a blood pressure of 112/71 mmHg, pulse of 101 beats/minute, and respiratory rate of 19 breaths/minute. Pulmonary examination was notable for crepitations in the left lung on auscultation, while the right lung was clear. Abdominal examination showed a softly distended, non-tender abdomen with audible bowel sounds and no signs of hepatosplenomegaly. Additionally, there were no signs of clubbing or swelling in the extremities. 

Laboratory findings 

Initial laboratory results on admission demonstrated white blood count 11.90 × 10^3/L (reference range: 4.50-10.50 x 10^3), neutrophils 8.85 x 10^3/L (reference range: 2.03-7.35 x 10^3), erythrocyte sedimentation rate 42 mm/hr (reference range: 0-13 mm/hr), C-reactive protein 93 mg/L (reference: <5 mg/L), total protein 5.5 g/dL (reference range: 5.7-8.2 g/dL), albumin 3.0 g/dL (reference range: 3.2-4.8 g/dL), lactate dehydrogenase 199 U/L (reference range: 120-246 U/L), hemoglobin 11.2 g/dL (reference range: 12.0-16.0 g/dl), hematocrit 34.5% (reference range: 36.0-48.0%), mean corpuscular volume 81.6 fl (reference range: 80.9-99.9 fl), and platelet count 744 x 10^3 (reference range: 150-450 x 10^3). These laboratory findings indicate an inflammatory process with some degree of reactive response. 

Subsequent laboratory testing during the patient's hospital stay showed positive Epstein-Barr Virus (EBV) Capsid Antigen immunoglobulin G and EBV Nuclear Antigen. Stool tests for Clostridium difficile toxin, Shigella/Enteroinvasive Escherichia coli, Salmonella, Escherichia coli, Shiga toxin, Norovirus Genogroup 1 (GI) and Genogroup II (GII), and Campylobacter jejuni/coli were all negative. Viral hepatitis panels for A, B, and C were also negative.

CT imaging 

**Figure 1:**
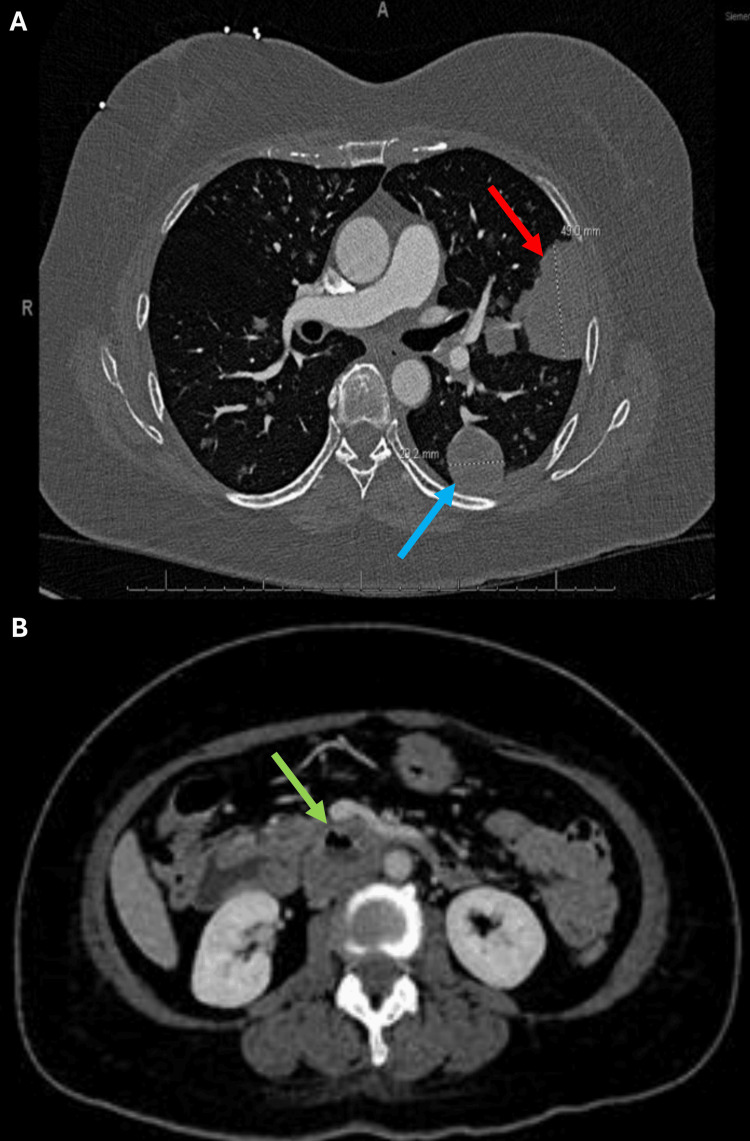
CT of the Chest, Abdomen, and Pelvis A: 4.9 cm left upper lobe peri-fissural mass (red arrow) and a 2.9 cm left lower lobe subpleural mass (blue arrow). B: A centrally necrotic 4.3 cm transverse duodenal mass (green arrow) narrowing the superior mesenteric vein and inferior vena cava.

A CT scan of the chest with intravenous (IV) contrast revealed several pulmonary masses, including a 4.9 cm left upper lobe peri-fissural mass and a 2.9 cm left lower lobe subpleural mass. A CT scan of the abdomen/pelvis with and without IV contrast showed a centrally necrotic 4.3 cm transverse duodenal mass narrowing the superior mesenteric vein and inferior vena cava (Figure 1).

Esophagogastroduodenoscopy/colonoscopy 

**Figure 2:**
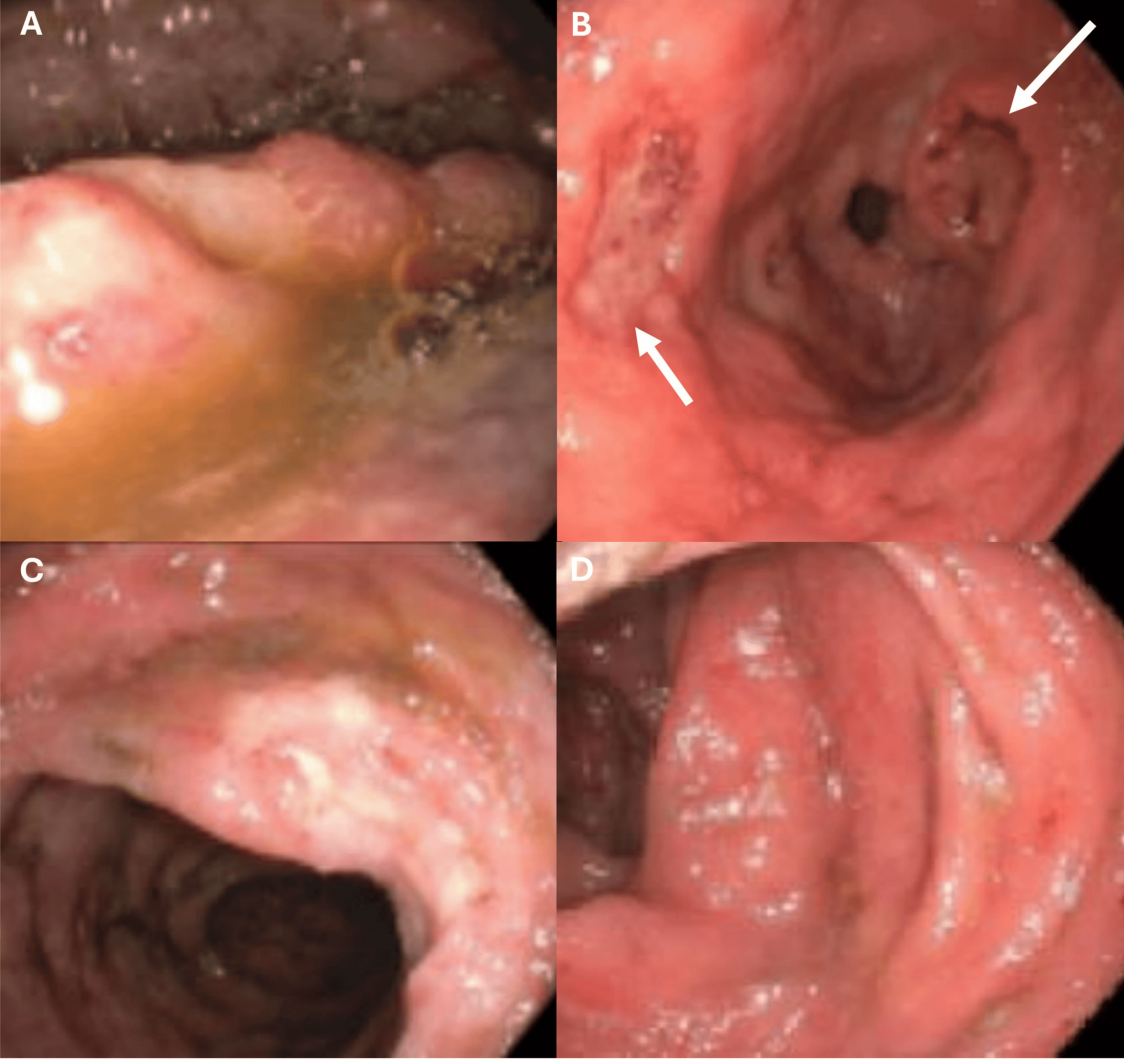
Esophagogastroduodenoscopy and Colonoscopy A: A localized nodular mucosa in the second portion of the duodenum. B: Antral and pre-pyloric ulcers (white arrows). C and D: Inflamed mucosa in the ascending and transverse colon.

Esophagogastroduodenoscopy revealed localized nodular mucosa in the duodenum, along with antral and pre-pyloric ulcers. Colonoscopy revealed inflamed mucosa throughout the entire colon (Figure 2).  Biopsies were performed on the duodenum, cecum, ascending colon, transverse colon, descending colon, sigmoid colon, and rectum.

Immunohistochemical analyses**

**Figure 3:**
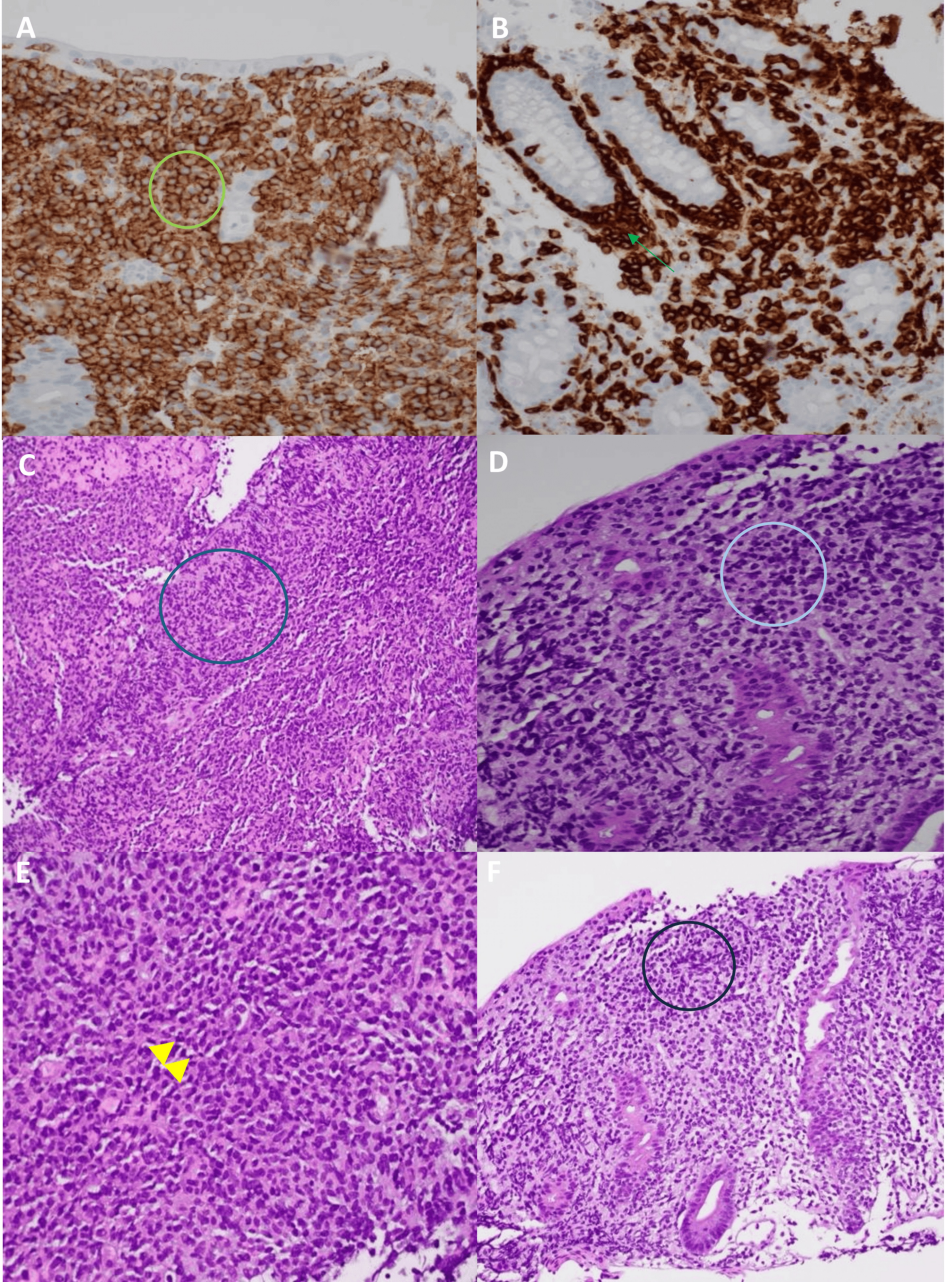
Immunohistochemical Analyses of a Biopsy Sample From the Cecum and Lung A: Immunohistochemical stain of the cecum with CD56 at high magnification (400X) demonstrating neoplastic T-cells involving surface epithelial cells (green circle). B: Immunohistochemical stain with CD3 at high magnification (400X) view of neoplastic CD3 positive T-cells diffusely infiltrating the base of intestinal crypts (green arrow). C: Hematoxylin-Eosin stain with low magnification (200X) view of neoplastic T-cells infiltrating lung parenchyma with no recognizable lung tissue (blue circle). D: Hematoxylin-Eosin stain of the lung at high magnification (400X) view of neoplastic T-cells that are pleomorphic and intermediate to large in size (light blue circle). E: Hematoxylin-Eosin stain of the cecum at low magnification (200X) view of neoplastic T-cells diffusely infiltrating lamina propria and involving crypts and surface epithelium (yellow arrowheads). F: Hematoxylin-Eosin stain of the cecum at high magnification (400X) of neoplastic T-cells with clear cytoplasm on the mucosal surface epithelium (dark blue circle).

Immunohistochemical analyses of the colon biopsy revealed neoplastic T-cell involvement with positive staining for CD3, CD7, CD8, CD56, CD103, TCR-delta, Granzyme B, TIA-1, and SYK. The neoplastic CD3 and CD56 positive T-cells were seen involving surface epithelial cells and the base of intestinal crypts. Hematoxylin-eosin stain of the lungs showed intermediate to large neoplastic T-cells infiltrating the lung parenchyma with no recognizable tissue. Given the immunomorphological findings and the absence of celiac disease history, a diagnosis of MEITL was favored (Figure 3).

Outcome

The patient’s preference was to receive chemotherapy at a specialized tertiary cancer center where she received three courses of ICE chemotherapy that consisted of ifosfamide, carboplatin, and etoposide, followed by two courses of high-dose methotrexate due to central nervous system (CNS) involvement later diagnosed at that center. Both positron emission tomography and MRI-brain with IV contrast performed at the center demonstrated excellent responses.

## Discussion

MEITL is a rare and aggressive form of primary T-cell lymphoma that is derived from intestinal epithelial T-cells. It accounts for less than 5% of primary malignant lymphomas of the digestive tract [3]. In 2008, the World Health Organization (WHO) classified enteropathy-type T-cell lymphoma into two types; Enteropathy-associated T-cell lymphoma type I (EATL type I) and enteropathy-associated T-cell lymphoma type II (EATL type II) [1]. In 2016, the WHO revised the classifications of lymphoid neoplasms and renamed EATL type I to EATL, which is closely linked to celiac disease and primarily affects individuals of Northern European origin. EATL type II was renamed to MEITL, which shows no association with celiac disease and affects Asians and Hispanic populations [1].

MEITL is recognized as a distinct entity due to its unique clinical, histopathological, and molecular features. Histologically, it is monomorphic and typically positive for CD8, CD56, and MATK [1]. Genetically, MEITL is frequently associated with SETD2 gene alterations and activating mutations in the JAK/STAT signaling pathway, primarily STAT5B, JAK3, and JAK1 [4]. Our patient exhibited somatic mutations in JAK3 and SETD2, consistent with a prior study [4], which are associated with increased clinical risk.

Despite its distinct features, MEITL shares pathophysiological similarities with other hematologic diseases, including chronic myeloid leukemia (CML). Both conditions involve clonal hematopoiesis, JAK-STAT pathway activation, immune dysregulation, and chronic inflammation-driven oncogenesis, leading to disease progression and treatment resistance. Additionally, both MEITL and CML can present with pancytopenia and hypersplenism which may contribute to diagnostic challenges. Notably, hypersplenism in CML has been associated with rare but fatal complications such as spontaneous splenic rupture, highlighting the need for vigilant monitoring in patients with hematologic malignancies and splenomegaly [5]. 

The diagnosis of MEITL remains challenging as many patients exhibit non-specific symptoms including chronic diarrhea, weight loss, chronic abdominal discomfort, and uncommonly, life-threatening gastrointestinal bleeding [6,7]. To our knowledge, extraintestinal manifestations are rare and there have only been a few reported cases of lung and brain involvement [3,8,9,10]. Suzuki et al. reported involvement of multiple nodules with cavitation and thick-walled cysts in the bilateral lungs [9]. In our patient, multifocal pulmonary masses were present, along with subsequent CNS involvement.

Lymphoma involvement in the pulmonary system can arise through various mechanisms including primary pulmonary origin, hematogenous metastasis from another primary site, or direct invasion from adjacent nodal lymphoma. Notably, our patient initially exhibited respiratory symptoms, including shortness of breath and cough, preceding gastrointestinal manifestations. During her hospital course, her diarrhea was initially attributed to recent antibiotic use and contrast exposure. Thus, early bronchoscopy and biopsy in cases with unexplained pulmonary symptoms may aid in earlier detection of MEITL.

On gross examination of the intestines, MEITL is often characterized by multiple circumferential ulcers accompanied by gut perforation and necrosis [8]. In contrast, our patient’s colonoscopy showed only diffuse areas of mildly inflamed mucosa throughout the colon with no evidence of perforation, necrosis, or ulceration. A previous study on MEITL prognosis identified elevated serum LDH and CRP levels as significant risk factors for poor outcomes [9]. In our patient’s case, LDH levels remained within normal limits, while CRP was markedly elevated. This could still be indicative of disease activity and systemic involvement. Although elevated LDH is a known risk factor, elevated CRP alone may still signal a more aggressive clinical course.

MEITL carries a poor prognosis, with reported overall survival of approximately seven months and progression-free survival of one month [11]. This poses significant challenges in managing and treating this aggressive disease. Currently, there is no standardized treatment strategy; however, one study explored anthracycline-based chemotherapy [12]. Unfortunately, many patients in this trial experienced treatment failure due to disease progression or toxicity. Despite this, the study also introduced an induction regimen combining ifosfamide, etoposide, and epirubicin alternating with immediate-dose methotrexate, which showed improved remission rates and reduced mortality, similar to the treatment that our patient was receiving.

## Conclusions

MEITL is a rare and aggressive lymphoma and is associated with a poor prognosis. This disease often poses diagnostic challenges due to its nonspecific manifestations including abdominal pain, chronic diarrhea, weight loss, and extraintestinal symptoms. Our patient’s presentation with initial pulmonary symptoms, subsequent gastrointestinal involvement, and delayed CNS diagnosis emphasizes the difficulty in diagnosis. Despite recent advancements in treatment including the incorporation of ICE regimens and methotrexate, outcomes remain unfavorable for most patients. This case underscores the need to recognize MEITL not only in its classic gastrointestinal presentation but also in atypical cases across diverse ethnic populations. Future efforts should prioritize establishing standardized diagnostic criteria and improving early detection strategies to enable timely intervention. Additionally, further research into novel targeted therapies and immunomodulatory treatments may offer new hope in improving patient prognosis. Our case illustrates the critical need for heightened awareness of atypical MEITL presentations to ensure earlier diagnosis and more effective strategies for this aggressive malignancy. 
